# Process, structural, and outcome quality indicators of nutritional care in nursing homes: a systematic review

**DOI:** 10.1186/s12913-018-2828-0

**Published:** 2018-01-26

**Authors:** Chiara Lorini, Barbara Rita Porchia, Francesca Pieralli, Gugliemo Bonaccorsi

**Affiliations:** 1 Department of Health Science, University of Florence, viale GB Morgagni 48, 50134 Florence, Italy; 2 School of Specialization in Hygiene and Preventive Medicine, University of Florence, viale GB Morgagni 48, Florence, Italy

**Keywords:** Malnutrition, Nutritional care, Structural indicators, Process indicators, Nursing homes

## Abstract

**Background:**

The quality of nursing homes (NHs) has attracted a lot of interest in recent years and is one of the most challenging issues for policy-makers. Nutritional care should be considered an important variable to be measured from the perspective of quality management. The aim of this systematic review is to describe the use of structural, process, and outcome indicators of nutritional care in NHs and the relationship among them.

**Methods:**

The literature search was carried out in Pubmed, Embase, Scopus, and Web of Science. A temporal filter was applied in order to select papers published in the last 10 years. All types of studies were included, with the exception of reviews, conference proceedings, editorials, and letters to the editor. Papers published in languages other than English, Italian, and Spanish were excluded.

**Results:**

From the database search, 1063 potentially relevant studies were obtained. Of these, 19 full-text articles were considered eligible for the final synthesis. Most of the studies adopted an observational cross-sectional design. They generally assessed the quality of nutritional care using several indicators, usually including a mixture of many different structural, process, and outcome indicators. Only one of the 19 studies described the quality of care by comparing the results with the threshold values. Nine papers assessed the relationship between indicators and six of them described some significant associations—in the NHs that have a policy related to nutritional risk assessment or a suitable scale to weigh the residents, the prevalence or risk of malnutrition is lower. Finally, only four papers of these nine included risk adjustment. This could limit the comparability of the results.

**Conclusion:**

Our findings show that a consensus must be reached for defining a set of indicators and standards to improve quality in NHs. Establishing the relationship between structural, process, and outcome indicators is a challenge. There are grounds for investigating this theme by means of prospective longitudinal studies that take the risk adjustment into account.

## Background

With the increase in life expectancy and the prevalence of disabilities and comorbidity related to aging, nursing homes (NHs) now play an increasingly important role.

The quality of NHs has attracted a lot of interest in recent years and is one of the most challenging issues for policy-makers. In the NH sector, poor quality represents an issue of public concern and discussions are taking place to address it [1–4]. The quality of care in NHs is a multidimensional construct that is difficult to define and assess. According to Donabedian’s framework [5], quality is a function of three domains: structure, process, and outcome. Structure is defined by the attributes of the settings in which care is provided, process by the activities of the care-giving practitioners, and outcome by the change in the health status of the patient. Within these three domains, the quality of care can be measured by using the structure, process, and outcome quality indicators.

The use of structural and process indicators for quality management offers several advantages — they are generally easy to measure and interpret and the collected data are often routinely available. However, they might not reflect the level of the quality of care; structural and process indicators indicate the attributes of the NH and what is being done (or is supposed to be done), but they do not automatically translate into a higher quality of care or better outcomes. Therefore, they are ‘necessary but not sufficient’ characteristics and do not necessarily indicate the appropriateness of what is being done [6, 7]. Moreover, the NH context is complex and very little knowledge translation has been carried out to date [8–10]. Outcome indicators overcome these limitations and are considered to be more closely related to quality. However, they are influenced by the risk level of elderly patients—primarily due to their health status—as well as by the quality of the care process. For these reasons, outcome indicators have to be risk-adjusted [7, 11].

Moreover, in order for structural and process indicators to be valid for NHs in terms of other care settings, they must first demonstrate the ability to generate a better outcome [6]. Specifically, they should be associated with and influence the outcome indicator, for example in terms of variation over time.

These unresolved issues and limitations in the use and interpretation of quality indicators have led to difficulties in assessing the real influence of the structural and process indicators on the prediction of the outcome indicators. Difficulties have also arisen, in general, in the evaluation of the effectiveness of quality indicators and quality systems for improving the quality of care, health status, and quality of life in NHs [12–15].

Malnutrition and unintentional weight loss in the NHs are major issues because of their high prevalence, serious health consequences, and related healthcare costs [16–20]. Recent studies estimate that 20% of NH residents suffer from some form of malnutrition, the prevalence of which ranges between 1.5 and 66.5%, depending on the definition [17]. Moreover, malnutrition can influence the health status, leading to clinical complications such as impaired immune response, depression, pressure ulcers, falls, and even death [18].

The causes of malnutrition and weight loss in elderly people living in long-term care facilities can be classified as either individual (age, comorbidity) or organizational [21, 22]. For many elderly adults in NHs, aging is accompanied by a progressive physiological and medical decline, which leads to nutritional vulnerability. This in turn can create a progressive feeding dependency. Many organizational factors can negatively affect the assumption of nutritionally adequate diet for such people, thus increasing the likelihood of malnutrition and weight loss. Therefore, nutritional care (i.e. the substances, procedures, and setting involved in ensuring the proper intake and assimilation of nutrients) must be considered an important variable that should be measured from the perspective of quality management by using the related structural, process, and outcome indicators [12, 22–26].

The aim of this review is to describe the state of the art with regard to:

1. the use of quality indicators of nutritional care in NHs;
2. the relationship between structural, process, and outcome indicators of nutritional care in NHs.

## Methods

The literature search was carried out in four databases—Pubmed, Embase, Scopus, and Web of Science—and was completed with a manual search on the basis of the references given in the selected papers.

While performing the research, a temporal filter was applied in order to select papers published in the last 10 years. Databases were last accessed on 18 February 2016.

The search strategies used in each database are reported in Table 1.

**Table 1:** Search strategies of systematic review

DATABASE	Search strategy
Pubmed	((((((“Quality Assurance, Health Care”[Mesh]) OR “Quality Improvement”[Mesh]) OR “Quality Indicators, Health Care”[Mesh]) OR “Health Care Quality, Access, and Evaluation”[Mesh])) AND “last 10 years”[PDat]) AND ((“Malnutrition”[Mesh] OR “nutritional care” OR “weight loss”) AND “last 10 years”[PDat]) AND ((“Nursing Homes”[Mesh] OR “Long-Term Care”[Mesh]) AND “last 10 years”[PDat])
Embase	quality OR indicator* OR assurance OR ‘health care’/exp. AND (‘malnutrition’/exp. OR ‘nutritional care’ OR ‘weight loss’/exp) AND ‘nursing home*
Scopus	(((quality OR indicator* OR assurance OR “health care”) AND (malnutrition OR “nutritional care” OR “weight loss”) AND (nursing home*)))
Web of Science	(((quality OR indicator* OR assurance OR “health care”) AND (malnutrition OR “nutritional care” OR “weight loss”) AND (nursing home*)))

Two reviewers independently selected papers based on the inclusion criteria. Disagreements were resolved through a consensus meeting in the presence of a third reviewer.

In order to be included, papers had to examine both care quality and nutritional care in the specific setting of NHs; moreover, they had to respond to the aims of this study, namely to describe the use of quality indicators of nutritional care in NHs and/or to assess the relationship between structural, process, and outcome indicators of nutritional care in NHs. All types of studies were included, with the exception of reviews, conference proceedings, editorials, and letters to the editor.

Papers published in languages other than English, Italian and Spanish were excluded.

**Fig. 1:**
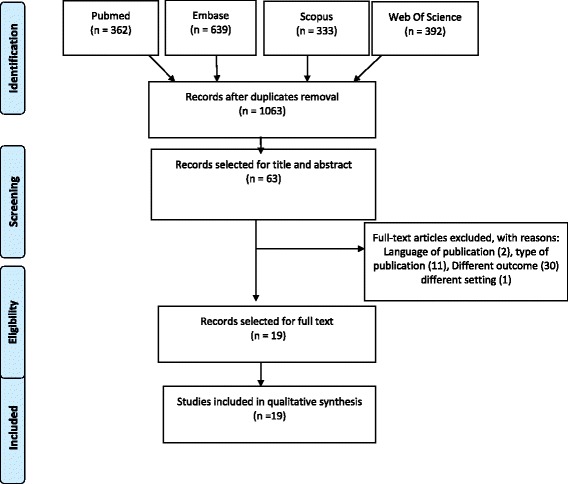
Flow diagram of the study selection [58]

Figure 1 summarizes the selection process of the articles.

## Results

From the database search, 1063 potentially relevant studies were obtained and screened for the presence of all inclusion criteria. Of the 63 studies selected on the basis of title and abstract, 44 were excluded: two because of language of publication, 11 for type of publication (four conference proceedings, three narrative reviews, three editorials, and one letter to the editor), 30 for outcomes (24 not concerning quality aspects, four not reporting quality indicators, and two not concerning nutritional aspects), and one for setting. Ultimately, 19 full-text articles were considered eligible for the final synthesis (Fig. 1).

Table 2 shows the main characteristics of each of the selected papers, including year of publication, country, setting, number of participants, type, and aim of the study. Most of the studies were conducted in the USA or Europe and adopted an observational cross-sectional design. One study [27] combined the Delphi method with an observational design. In two papers, the authors conducted a before/after analysis [28, 29].

**Table 2:** Main characteristics of selected studies

1st Author, Year of publication	Country	Setting/ n. participants	Type of study	Aim of the study
Bonaccorsi, 2015 [35]	Italy	67 NHs; 2395 participants	Cross-sectional survey	To describe the quality indicators of nutritional care in older residents in a sample of NHs in Tuscany, Italy, and to evaluate the predictors of protein-energy malnutrition risk.
Dyck, 2007 [39]	USA	2948 NHs for malnutrition; 364,339 residents	Cross-sectional analysis of two data sets	To examine the relationships between nursing staffing and the nursing home resident outcome on weight loss and dehydratation .
Halfens, 2013 [30]	The Netherlands, Austria, Switzerland	211 hospitals (20,232 patients); 165 NHs (6969 residents)	Cross-sectional multicentre study.	To measure care problems (including malnutrition) in terms of prevalence rates, prevention, treatment, and quality indicators in healthcare organizations in the Netherlands, Austria, and Switzerland.
Hjaltadottir, 2012 [27]	Iceland	Panel for Delphi method: 12 experts; 47 NHs (2247 participants)	Two rounds Delphi study and observational study	To determine upper and lower thresholds of Minimum Data Set quality indicators for Icelandic NHs.
Hurtado, 2016 [40]	USA	30 NHs	Prospective ecological study	To examine whether quality of care in NHs was predicted by schedule control (workers’ ability to decide work hours), independent of other staffing characteristics.
Lee, 2014 [41]	USA	195 NHs	Cross-sectional analysis of five data sets	To examine the association of registered nurse staffing hours and five quality indicators, including process and outcome measures.
Meijers, 2009 [59]	The Netherlands	50 hospitals, 90 NHs, 16 care homes, and 20,255 participants	Cross-sectional multicentre study	To investigate screening, treatment, and other quality indicators of nutritional care in Dutch healthcare organizations.
Meijers, 2014 [36]	The Netherlands	74 Care homes (41 participated four times,33 five times); 26,046 participants (2007–2011)	Cross-sectional study	To analyse the trend of malnutrition prevalence rates between 2007 and 2011 in Dutch care homes and the effect of process and structural indicators on malnutrition prevalence rates.
Moore, 2014 [31]	Australia	Four Residential Aged Care (RAC)	Cross-sectional study	To explore relationships among the Victorian Public Sector RAC Services quality indicators and other demographic and health-related issues.
Rantz, 2009 [29]	USA	492 NHs	Before-after observational study	To present and discuss the evaluation of the Quality Improvement Program of Missouri in 2006, using some outcome indicators.
Schönherr, 2012 [32]	Austria	18 NHs (1487 participants); 18 hospitals (2326 participants)	Multicentre cross-sectional study	To describe and compare structural and process indicators of nutritional care in Austrian hospitals and NHs.
Shin, 2015 [42]	Korea	150 NHs	Cross-sectional study	To investigate the relationship between nurse staffing and quality of care in NHs in Korea.
Simmons, 2006 [28]	USA	1 NHs (48 beds)	Before-after observational study	To train long-term care staff in conducting continuous quality improvement (CQI) related to nutritional care.
Simmons, 2007 [44]	USA	7 NHs	Cross-sectional study	To assess the impact of Paid Feeding Assistant (PFA) programmes on feeding assistance care process quality.
Van Nie, 2014 [37]	The Netherlands, Germany and Austria	214 NHs 19,876 residents	Multicentre cross-sectional study	To identify structural quality indicators of nutritional care that influence the outcome of quality of care in terms of prevalence of malnutrition and effect of possible differences between malnutrition prevalence in Dutch, German, and Austrian NHs.
van Nie-Visser, 2011 [33]	The Netherlands and Germany	151 NHs, 10,771 participants	Multicentre cross-sectional study	To investigate possible differences in malnutrition prevalence rates in Dutch and German NHs, as well as in structural and process indicators for nutritional care
van Nie-Visser, 2014 [34]	The Netherlands, Germany and Austria	214 NHs; 19,876 residents	Multicentre cross-sectional study	To investigate possible differences in malnutrition prevalence rates in Austrian, Dutch, and German NHs, as well as in structural and process indicators for nutritional care; to investigate whether resident characteristics influence possible differences in malnutrition prevalence between countries.
van Nie-Visser, 2015 [38]	The Netherlands, Germany and Austria	214 NH; 22,886 participants,	Multicentre cross-sectional study	To explore whether structural quality indicators for nutritional care influence malnutrition prevalence in Dutch, German, and Austrian NHs
Werner, 2013 [43]	USA	16,623 NHs	Cross- sectional study using 2 data sets	To test how changes in NH processes improve outcomes of care.

Seven studies only aimed to measure the prevalence of malnutrition/weight loss (as outcome indicator) and the use of structural or process indicators [20, 27, 30–34]. Four others tried to assess both the prevalence of malnutrition and the relationship among the quality indicators [35–38]. Five only assessed the relationship between indicators (without describing their prevalence/use) [39–43], and three examined the effect of nutritional care interventions on outcome indicators [28, 29, 44].

With regard to the collection of information, the most commonly used instruments were the standardized *Landelijke Prevalentiemeting Zorgproblemen* (LPZ) questionnaire, the Minimum Data Set (MDS), and the Online Survey, Certification, and Reporting (OSCAR). LPZ is more widely used in European countries and aims to assess malnutrition prevalence. MDS and OSCAR are more common in the American context—the former predicts unplanned weight loss while the latter includes facility-reported data on residents’ characteristics. In some other studies [28, 35, 42, 44], ad hoc instruments were used. In one of them, the ad hoc instrument was improved on the basis of a literature analysis [35]. Hurtado et al. [41] used both standardized instruments and ad hoc questionnaire.

The selected papers show heterogeneity in the considered quality indicators, particularly the structural and process indicators. As regards the outcome indicators, the authors considered the risk of malnutrition (according to Malnutrition Universal Screening Tool), weight loss (according either to MDS or VPSRAC - Victorian Public Sector Residential Aged Care Services - definition), and malnutrition prevalence (according to LPZ questionnaire) (Table 3).

**Table 3:** Quality indicators of nutritional care reported in the selected papers

1st Author, Year of publication	Instruments for collecting data on quality indicators	Structural/process indicators	Outcome indicators
Bonaccorsi, 2015 [35]	Ad hoc instruments (questionnaire/direct observation)	Structural indicators	Prevalence of subjects with medium to high risk of malnutrition, according to MUST.
		Type of scales used to weigh residents	
		Employment of dietitians and type of consultation	
		Number of operators assigned to manage the administration of meals in a specific day	
		Process indicators	
		Use of a nutrition screening tool	
		Presence of protocols/guidelines for weight assessment	
		Presence of protocols or guidelines for administration of food	
		Assessment of dysphagia	
Dyck, 2007 [39]	MDS; OSCAR	Staffing hours:	Weight loss^a^
		- RN hours per resident per day	
		- LPN hours per resident per day	
Halfens, 2013 [30]	LPZ	Not described	Malnutrition prevalence^b^
Hjaltadottir, 2012 [27]	MDS	–	Weight loss^a^
Hurtado, 2016 [40]	Nursing Home Compare/MDS; ad hoc questionnaire	Schedule control (from ad hoc questionnaire):	Weight loss^a^
		- to choose when to take day off or vacation	
		- to choose when to start/end each work day	
		- to choose when to take a few hours of break	
		- to decide how many hours to work each day	
Lee, 2014 [41]	MDS; the Colorado state inspections	RN staffing hours (from the Colorado state inspections data)	Weight loss^a^
Meijers, 2009 [59]	LPZ	Institutional level	Malnutrition prevalence^b^
		Availability of an up-to-date protocol/guideline on malnutrition prevention and treatment	
		Auditing of protocol/guideline for malnutrition prevention and treatment	
		Availability of malnutrition advisory teams	
		Multiple dietitians available in the institution	
		Malnutrition education (prevention and treatment) given by malnutrition specialist within the last two years	
		Ward level	
		Trained malnutrition specialist working on the ward	
		Control of use of prevention and treatment guidelines	
		Policy to measure weight at admission	
		Documentation of malnutrition interventions	
		Correct mealtime ambience	
Meijers, 2014 [36]	LPZ	Structural indicators	Malnutrition prevalence^b^
		Institutional level	
		There is an agreed protocol/guideline for the prevention and/or treatment of malnutrition within the institution.	
		There is an advisory committee for malnutrition at the institution or department level.	
		There is someone within the institution who is responsible for updating and ensuring that the necessary attention is devoted to the malnutrition protocol.	
		Over the last two years, a refresher course and/or a meeting was organized for caregivers, which was/were specifically devoted to the prevention and treatment of malnutrition within the institution.	
		Ward level	
		There is at least one person/specialist in the department/basic care unit/team who is specialized in the area of malnutrition.	
		Work in the department/basic care unit/team is done in a controlled fashion or in accordance with the malnutrition protocol/guideline.	
		Upon admission, every resident is weighed as a part of standard procedure.	
		The nutritional status is screened upon admission.	
		The care file/care plan specifies the activities that must be implemented for residents who are at risk of malnutrition.	
		The department has a policy on when and how to measure weight.	
		Process indicators	
		Assessment of the nutritional status by a validated screening instrument	
		Weight monitoring in a controlled fashion	
		Dietitian consultation	
		Use of nutritional treatment	
Moore, 2014 [31]	VPSRACS; data routinely collected in the facilities included in the study	–	Weight loss^c^
Rantz, 2009 [29]	MDS	Not described (QIPMO—nurse site visits to suggest how to improve quality of care)	Weight loss^a^
Schönherr, 2012 [32]	LPZ	Structural indicators:	Malnutrition prevalence^b^
		Guideline for prevention and treatment	
		Auditing of guideline	
		Advisory committee for malnutrition	
		Updating of guideline	
		Criteria for determining malnutrition	
		Employment of dietitians	
		Refresher course for caregivers	
		Information brochure	
		Standard policy for handover	
		Process indicators	
		Assessment of weight	
		Use of nutritional screening tool	
		Assessment of weight over time	
		Use of clinical view	
		Use of biochemical parameters	
		Dietitian consulted	
		Energy- and protein-enriched diet	
		Energy-enriched snack	
		Oral nutritional support	
		Enteral nutrition	
		Parenteral nutrition	
		Texture-modified diet	
		Fluid 1–1.5 L/d	
		No interventions owing to palliative policy	
Shin, 2015 [42]	Ad hoc instruments (questionnaire-interviews)	Nurse staffing, by type (RN, CNA, qualified care workers):	Weight loss^a^
		- hours per resident per day	
		- skill-mix hours per resident per day	
		- staff turnover	
Simmons, 2006 [28]	Ah hoc instruments (direct observation)	Feeding Assistance Care Process Measure:	–
		-% of residents who eat less than 50% of meal and receive less than one min of assistance.	
		-% of residents who eat less than 50% of meal and are not offered a substitute.	
		-% of residents who receive less than five min of assistance and a supplement.	
		-% of residents who are independent but receive physical assistance.	
		- % of residents who receive physical assistance without verbal cue.	
Simmons, 2007 [44]	Ah hoc instruments (direct observation)	Feeding Assistance Care Process Measure, by type of staff (CNAs, PFAs, no assistance from either type of staff):	–
		-% of residents who eat less than 50% of meal and receive less than one min of assistance.	
		-% of residents who eat less than 50% of meal and are not offered a substitute.	
		-% of residents who receive less than five min of assistance and a supplement.	
		-% of residents who are independent but receive physical assistance.	
		- % of residents who receive physical assistance without verbal cue.	
Van Nie, 2014 [37]	LPZ	Structural indicators	Malnutrition prevalence^b^
		Institutional level	
		There is an agreed protocol/guideline for the prevention and/or treatment of malnutrition within the institution.	
		Malnutrition-related work within the institution is carried out in a controlled fashion or in accordance with a malnutrition protocol/guideline.	
		There is a multidisciplinary advisory committee for malnutrition at the institutional or ward level.	
		There is someone within the institution who is responsible for updating and ensuring that the necessary attention is devoted to the malnutrition protocol.	
		Within the institution, criteria have been defined for determining malnutrition.	
		There are dietitians employed at the institution.	
		Over the past two years, a refresher course and/or a meeting has been organized for caregivers, which was specifically devoted to the prevention and treatment of malnutrition within the institution.	
		An information brochure about malnutrition is available at the institution for clients and/or family members.	
		Ward level	
		There is at least one nurse in the ward who is specialized in the area of malnutrition	
		Clients who are at risk of malnourishment or who are malnourished are discussed on the ward during multidisciplinary work consultations.	
		Work in the ward is conducted in a controlled fashion or in accordance with a malnutrition protocol/guideline.	
		At admission, every client is weighed as a part of standard procedure.	
		At admission, the height of each client is determined as a part of standard procedure.	
		The nutritional status is assessed at admission.	
		The care file includes an assessment as to each patient’s risk of malnutrition.	
		The care file/care plan specifies the activities that must be implemented for clients who are at risk of malnutrition.	
		In case of (expected) malnutrition, a protein- and energy-enriched diet is provided in the ward as a part of standard procedure.	
		Every client who is malnourished (or is at risk for becoming so) and his or her family receive an informational brochure about malnutrition.	
		The ambience at mealtimes is taken into account within the ward.	
		The care file includes the intake for each client.	
		The ward has a weight policy.	
van Nie-Visser, 2011 [33]	LPZ	Structural indicators	Malnutrition prevalence^b^ and prevalence of subjects with risk of malnutrition.
		Institution level	
		Prevention and treatment protocol/guideline	‘At risk of malnutrition is defined as meeting one or more of the following criteria: (1) BMI 21–23.9 kg/m^2^, (2) not eaten or hardly eaten anything for three days or not eaten normally for more than a week.
		Malnutrition advisory team	
		Auditing of protocol/guideline	
		Dietitians employed in institution	
		Education on malnutrition prevention and treatment in last 2 years	
		Information brochure available for client or family	
		Ward level	
		Person specialized in malnutrition on unit	
		Control of use of prevention/treatment guideline	
		Measurement of weight at admission	
		Interventions on malnutrition stated in patient file	
		Optimal mealtime ambience provided at dinner	
		Process indicators	
		Assessment of weight	
		Use of nutritional screening tool	
		Weight history	
		Use of clinical view	
		Use of biochemical parameters	
		Energy- and protein-enriched diet	
		Energy-enriched snacks between meals	
		Oral nutritional supplements	
		Tube feeding	
		Parenteral feeding	
		Fluid 1–1.5 L/d	
		No interventions	
		Palliative policy	
van Nie-Visser, 2015 [38]	LPZ	See above (….)	Malnutrition prevalence^b^
van Nie-Visser, 2014 [34]	LPZ	–	Malnutrition prevalence^b^
Werner, 2013 [43]	MDS/Nursing Home Compare; OSCAR	-% of residents receiving tube feeds	Weight loss^a^
		-% of residents receiving mechanically altered diets	
		-% of residents with assisted eating devices	

*MUST* Malnutrition Universal Screening Tool

*MDS* Minimum Data Set

*LPZ* Landelijke Prevalentiemeting Zorgproblemen (In Dutch)

*VPSRACS* Victorian Public Sector Residential Aged Care Services

*OSCAR* Online Survey, Certification, and Reporting

*ARF* Area Resource File

*RN* Registered Nurse

*LPN* Licensed Practical Nurse

*CNA* certified nursing assistant

*QIPMO* Quality Improvement Program of Missouri

*PFA* Paid Feeding Assistant

^a^loss of 5% or more in the last months or loss of 10% or more in the past six months, as defined in MDS

^b^(1) BMI ≤ 18.5 kg/m2(age 18–65 years) or BMI ≤ 20 kg/m2 (age > 65 years), and/or (2) unintentional weight loss (more than 6 kg in the previous six month or more than 3 kg in the last month) and/or (3) no nutritional intake for three days or reduced intake for more than 10 days combined with a BMI between 18.5–20 kg/m2 (age18–65 years) or between 20 and 23.9 kg/m2(age > 65 years)

^c^loss of ≥3 kg over three months, or any unplanned weight loss for each consecutive month of the quarter

Of the19 selected papers, nine studies [29, 35–40, 42, 44] examined the influence of structural and process indicators on the outcome indicators (Table 4).

**Table 4:** Relationship between structural, process and outcome indicators of nutritional care

1st Author, Year of publication	Risk adjustment	Main results
Bonaccorsi, 2015 [35]	Age, gender, the Barthel Index score, the Pfeiffer test score, the EBS score, where the subject consumed lunch on the day of the survey	Among the process and structural indicators included in the study, the only one with a role in predicting malnutrition was the availability of a scale suitable for weighing residents even in the case of mobility restriction (chair or platform scale).
Dyck, 2007 [39]	Residents’ case-mix: end of life, depression, swallowing problem, renal failure, diabetes mellitus	Staffing hours affect weight loss: residents receiving at least three hours/day of nursing assistant care had a 17% decreased likelihood of weight loss.
Hurtado, 2016 [40]	High-risk residents’ adjustment at facility level (not described).	Schedule control was not associated with weight loss.
Meijers, 2014 [36]	NO	Only the interacted process indicators nutritional screening and oral nutritional supplementation were significant in influencing malnutrition prevalence rates over time. Structural indicators had no impact on the malnutrition prevalence over time.
Rantz, 2009 [29]	NO	‘At risk’ facilities (defined using quality indicators derived from MDS) accepting one or more visits improved weight loss quality indicators by 4%.
Shin, 2015 [42]	NO	Hours per resident per day, skill-mix hours per resident per day, and staff turnover are not statistically associated with weight loss.
Van Nie, 2014 [37]	NO	Five structural quality indicators influenced malnutrition prevalence in NH residents at the ward level: presence of at least one nurse in the ward specialized in the area of malnutrition; nutrition assessment upon admission; inclusion in the care file of the assessment as to the risk of malnutrition for each client; provision of a protein- and energy-enriched diet in case of (expected) malnutrition, in accordance with a standard procedure; inclusion in the care file of the intake for each client.
van Nie-Visser, 2015 [38]	NO	Two structural quality indicators of nutritional care at ward level influence malnutrition prevalence in NH residents: the policy that a care file should include the nutritional intake for each resident and the policy for ward having a weight measurement.
Werner, 2013 [43]	Data controlled for case-mix and for facility-level characteristics related to residents’ case-mix: • Age • Activity of Daily Living • Cognitive performance scale • % of residents who needs radiation therapy, chemotherapy, dialysis, intravenous therapy, respiratory treatments, tracheostomy care, ostomy care, suctioning, injections	The statistically significant improvement in weight loss indicator could not be explained by changes in the investigated measures of process of care (% of residents receiving tube feeds; % of residents receiving mechanically altered diets; % of residents with assisted eating devices).

*EBS* Eating Behaviour Scale, *MDS* Minimum Data Set, *OSCAR* Online Survey, Certification, and Reporting

In four of the studies [35, 39, 40, 43], an individual risk adjustment procedure was applied by using different variables and determining heterogeneity among the different studies. While five studies [29, 35–38] showed a significant association between some structural or process indicators and the outcome indicators, said association was found for different structural and process indicators.

## Discussion

In this review, we selected 19 papers in the aim of investigating the use of quality indicators of nutritional care in NHs. The selected papers assessed the quality of nutritional care in NHs in general by using several indicators, normally including a mixture of several structural, process, and outcome indicators. Most of the studies used standardized questionnaires or instruments to collect data on quality indicators, either routinely applied at a state level for mandatory reasons (MDS, Victorian Public Sector Residential Aged Care Services [VPSRACS]), or implemented as an annual measurement of malnutrition prevalence and structural quality indicators of nutritional care in the NHs that voluntarily decided to participate to the study (LPZ). As for the outcomes, different indicators were taken into account. However, weight loss was always included, although different combinations of time periods and cut-offs were considered for each instrument. It was evident that no consensus exists on the sets of indicators to be used, especially outcome indicators, even though only a few instruments were used to collect data. Nevertheless, according to our findings, the presence of nutritional screening and its inclusion in the care file, the availability and use of protocols on malnutrition prevention and treatment, mealtime assistance, and the use of nutritional treatment/supplements, all appear to be relevant indicators for nutritional care quality assessment. In any case, studies aimed at testing the reliability and validity of these indicators, as well as the outcome indicators, need to be developed in order to identify the best set of indicators for describing the quality of nutritional care in NHs. This is also in agreement with statements of other authors [45, 46].

Most of the papers aimed to describe the quality of nutritional care in NHs, at times also to compare the data in different geographical areas, settings, or time periods. However, they do not discuss the collected data in terms of good or poor quality with respect to a standard, with the exception of the paper by Hjaltadòttir et al. [27], in which the quality of care in Icelandic NHs was compared with the threshold values that had been determined in the same study. Thresholds for quality indicators could help guide and facilitate progress in the NHs’ quality of care, indicating the potentially poor or good quality of care and improvement goals [27]. Criteria and standards specify the expected outcome, and encourage the performer to progress towards fulfilling them. However, no internationally recognized comprehensive standards are available, although the laws and reforms of long-term care systems in many countries have also included aspects of quality assurance and improvement, such as the setting of minimum requirements as preconditions of licensing and contractual decisions for providers [2, 3]. The lack of internationally recognized standards can be attributed to the complexity of the context of long-term care and the fact that context and residents often differ considerably in the different NHs. Research on threshold values and standards for nutritional care should be encouraged, taking into account the specificity of the setting and the residents as well as the knowledge translation aspects [8].

The prevalence or risk of malnutrition is associated with aspects such as having a policy related to nutritional risk assessment (i.e. screening the subjects for malnutrition, weighing them, assessing and recording nutritional intake) or having suitable scales to weight the residents; when these aspects are present or used in NHs, the prevalence or risk of malnutrition is lower.

In two [36, 37] out of three [36–38] articles that investigated the provision of a protein- and energy-enriched diet, or the use of oral nutritional supplementation in case of (expected) malnutrition, this factor was found to be related to malnutrition. Malnutrition is more prevalent in institutions implementing this indicator. Therefore, providing an enriched diet or oral nutritional supplementation seems to be more of an intervention treatment than a preventive one. This hypothesis and the role of screening for malnutrition are both confirmed by the results of the study by Meijers et al. [36]—the only one in which a trend evaluation of the outcome indicator is carried out. In fact, according to the authors, structural screening is the most important indicator of a decrease in the prevalence of malnutrition. In NHs with a higher prevalence of malnutrition, more residents receive oral nutritional supplementation. While the provision of oral nutritional supplementation is associated with a gradual decrease in the prevalence of malnutrition, this drop is more pronounced if the use is lower, probably due to the fact that the group receiving less oral nutritional supplementation is probably in better health [36].

On the other hand, quality indicators related to the staff (i.e. employment of dieticians, malnutrition specialists, person in charge of the malnutrition protocol, or a multidisciplinary malnutrition advisory team, the organization of courses on malnutrition, and staff turnover) do not seem to affect the outcome indicators, with the exception of the ‘presence of at least one nurse in the ward specialized in the area of malnutrition’ in one of the papers by Van Nie et al. [37] and ‘receiving at least [three] hours/day of nursing assistant care’ in the study by Dyck et al. [39]. Consequently, the presence of a staff member with competencies in nutritional aspects and specific education or training is related to malnutrition risk in just one study, where it only concerns the presence of nurses with specific competencies in the area of malnutrition. This result is in line with the results of two reviews regarding staffing and the various aspects of the quality of care in NHs [13, 47].

Regarding the relationship between indicators, we have also included risk adjustment to control individual risk in our assessment, in order to generalize the results for residents with different levels of disabilities and comorbidities. The need for individual risk adjustment in the assessment of quality of care in NHs has emerged simultaneously with the growing attention to quality in healthcare, but only a few authors have considered this factor to avoid a biased use of quality indicators [11, 48]. Individual risk adjustment has yielded better results in terms of validity and comparability, since NH residents are quite dissimilar [3, 7, 49–54]. In our review, only four papers [35, 39, 40, 43] out of nine included risk adjustment, which could limit the comparability of the results. Risk adjustment should also be taken into account when identifying the thresholds for quality indicators in order to control the cut-off levels for individual risk.

Eight [35–40, 42, 43] of the nine articles describe the results obtained through a cross-sectional or ecological approach. One cross-sectional study includes a sample at the time of ascertainment, selected without any reference to exposure or health outcome (disease status or other condition of interest, such as risk of a disease). Exposure is determined simultaneously with the health condition, and different exposure subpopulations are compared with respect to their health status to assess correlation or association between exposure and outcome. Such studies have difficulty determining the chronological order of events (i.e. the beginning of the exposure and the onset of a health condition). Due to this limitation, it is not possible to work out whether an association between exposure and outcome demonstrated in a cross-sectional study underlies a cause-effect relationship. The same issue occurs for ecological studies in which the association or the correlation between exposure and health outcome is assessed using groups rather than individuals (the unit of analysis is the group, and the analysis is conducted without considering the individual level) [55]. Cross-sectional, ecological and other descriptive studies are often the initial tentative approaches to new events or conditions for generating a hypothesis for causation (‘hypothesis-generating’ studies). The etiologic hypothesis has to be tested through cohort, case-control, or experimental studies [56, 57]. Therefore, considering the study design of almost all articles included in this review, it is not possible to fully understand the type of relationship (i.e. etiologic or not) between process or structural indicators and outcome indicators.

One article [29] in the sample includes a before-after observational study aimed at evaluating a quality improvement programme that is not described in detail. As a result, when reading the paper it is not possible to understand whether the implemented measures would be able to foresee aspects concerning specific structural or process indicators.

## Conclusions

Our findings show that there is an open debate regarding the indicators that could be used to describe the quality of nutritional care in NHs. A consensus must be reached to define a set of indicators and a standard to improve the quality in NHs. For this purpose, studies aimed at testing the reliability and validity of the indicators are encouraged. Moreover, the relationships among structural, process, and outcome indicators are a matter of challenge. According to our results, while the prevalence or risk of malnutrition is associated with aspects such as having a policy related to nutritional risk assessment or having suitable scales to weigh the residents, these findings need to be confirmed. In conclusion, there are grounds for investigating this new theme by means of prospective longitudinal studies that also take the risk adjustment into account.

## Abbreviations

ARF: Area Resource File
BMI: Body Mass Index
CNA: Certified nursing assistant
EBS: Eating Behaviour Scale
LPN: Licensed Practical Nurse
LPZ: Landelijke Prevalentiemeting Zorgproblemen (In Dutch)
MDS: Minimum Data Set
MUST: Malnutrition Universal Screening Tool
NH: nursing home
OSCAR: Online Survey, Certification, and Reporting
PFA: Paid Feeding Assistant
QIPMO: Quality Improvement Program of Missouri
RN: Registered Nurse
VPSRACS: Victorian Public Sector Residential Aged Care Services
